# Perspective of Attending Physicians on the Use of Telemedicine in an Outpatient Arthroplasty Setting During the COVID-19 Pandemic

**DOI:** 10.1177/1556331620979984

**Published:** 2021-02-21

**Authors:** David A. Kolin, Kaitlin M. Carroll, Kevin Plancher, Fred Cushner

**Affiliations:** 1 Hospital for Special Surgery, New York, NY, USA; 2 Plancher Orthopaedics and Sports, New York, NY, USA

**Keywords:** telemedicine, orthopedic surgery, COVID-19, postoperative care

## Abstract

*Background*: During the worldwide COVID-19 pandemic, physicians had to improvise and adapt new ways to provide care to patients. *Purposes*: The purpose of this study was to assess physicians’ sentiments regarding telemedicine and its use in orthopedic practices. *Methods*: We performed a cross-sectional study of attending orthopedic physicians, the majority of whom integrated telemedicine into their practices from March to October 2020. A survey was sent to 517 physicians who had registered for an orthopedics conference. The survey included questions pertaining to various factors regarding telemedicine and each physician’s practice. *Results*: Of the 517 physicians who received the survey, 328 responded, for a 63.4% response rate. Of the 328 respondents, 84.1% did not use telemedicine in their practice prior to the COVID-19 pandemic. Even during the pandemic, the physicians most commonly responded that less than 5% of their practice was conducted by telemedicine (n *=* 103, 31.4%). The second most common response was that more than 20% of visits were done via telemedicine (n = 72, 22.0%); 43.0% of physicians noted that they would not use telemedicine technology in their practice after the pandemic, but 59.1% of physicians would be willing to do annual visits by telemedicine. Ability to examine the patient (2.0 ± 1.0) was rated worse, overall, than either the experience using the technology (3.2 ± 1.0) or the capacity to communicate with the patient (3.6 ± 1.0). *Conclusions*: Our survey of orthopedic surgeons demonstrates that while the use of telemedicine technology was minimal prior to the pandemic, its use was widely adopted during the pandemic. Nearly half of physicians said that they will continue to use telemedicine.

## Introduction

The coronavirus disease 19 (COVID-19) pandemic disrupted health care delivery worldwide and prompted new forms of health services. Because in-person contact became fraught with risk for both patients and health care providers, alternatives to in-person patient visits were explored to limit COVID-19 transmission. One mode of patient-physician communication that became particularly popular was telemedicine, defined broadly by the World Health Organization as the delivery of health care services at a distance using electronic means to improve health [18]. Telemedicine has been practiced since the 1980s, but was generally reserved for specific populations or individuals. Telemedicine use often pertained to military-related health consults and rural patient visits, where access to in-person health tends to be scarce. The adoption of telemedicine gained renewed interest in orthopedics in recent years but had not been broadly adopted prior to the pandemic. Consequently, the use of telemedicine during the pandemic was new for many physicians. Understanding telemedicine’s role in patient care and integration into physician practices was a learning experience during the worldwide pandemic.

While telemedicine is a critical asset during a pandemic, as it reduces risk of interpersonal viral transmission, a number of benefits may continue to prove useful, even after resolution of the pandemic [4]. Telemedicine reduces transportation time, allows for observation of patients in their home environment, and facilitates routine follow-up care [11,13]. In some cases, telemedicine may provide a more cost-effective means of treatment for both patients and physicians. The option of telemedicine can be highly beneficial for patients with reduced mobility, limited transportation options, or those who live in rural areas. Despite its strengths, telemedicine poses several limitations, including technological difficulties, inability to perform a thorough physical exam, and interstate licensure challenges [21].

Since the onset of the pandemic, telemedicine has been widely adopted in a range of settings. In 2016, approximately 10% of family physicians and pediatricians in the United States used telemedicine [12,15]. However, just 2 months into the COVID-19 pandemic, only 9% of physicians practicing in a family physician setting did not offer telemedicine services in their practice. Telemedicine has even been adopted by orthopedic surgeons. In one study investigating 168 orthopedic surgery programs participating in the Electronic Residency Application Service, 106 (63%) of the programs were offering telemedicine services to patients as early as April 2020 [16].

The purpose of this study was to assess physician sentiments regarding telemedicine and its use in orthopedic practices. We sought to explore physicians’ experiences with telemedicine in the outpatient arthroplasty setting and their thoughts about continued use of telemedicine following the COVID-19 pandemic.

## Methods

We performed a cross-sectional study of attending orthopedic physicians in the United States, the majority of whom integrated telemedicine into their practices from March 17 to October 6, 2020. We distributed a survey via GoogleForms (Fig. 1, see Supplemental Material) to 517 physicians who had registered for an orthopedics conference. The survey included questions addressing demographic factors, total number of total hip arthroplasties performed per year, number of total knee arthroplasties performed per year, physician satisfaction with telemedicine, and estimates of the proportion of visits conducted via telemedicine. The questions were designed to identify how the pandemic changed the physicians’ use of telehealth and to determine which follow-up appointments they would be willing to use telehealth for in the future. While the survey did not permit participants to skip any questions, there were options that allowed them to write in specific responses. The goal was to capture all data in a uniform manner but provide opportunity for participants to explain their perspectives. The final survey responses were reviewed for accuracy and appropriateness by a member of the research team.

Means and standard deviations (mean ± SD) were calculated for continuous variables. Totals and percentages were calculated for categorical variables. Free-text responses were assessed manually for any consistent themes among multiple respondents. All analyses were conducted using R software, version 4.0 (R Foundation for Statistical Computing, Vienna, Austria).

## Results

Of the 517 physicians who received the survey, 328 responded, for a 63.4% response rate. Physicians were most commonly in the 55- to 65-year age range (n = 123, 37.5%). Surgeons most commonly performed 0 to 49 (n = 197, 60.1%) total hip replacements per year and 0 to 49 (n = 144, 43.9%) total knee replacements per year (Table 1). Most of the physicians did not use telemedicine in their practice prior to the COVID-19 pandemic (n = 276, 84.1%) (Table 2). Even during the pandemic, physicians most commonly responded that <5% of their practice was conducted by telemedicine (n = 103, 31.4%). However, the second most common response was that >20% of visits were completed via telemedicine (n = 72, 22.0%). Nearly half of physicians surveyed (n = 141, 43.0%) noted that they would not use telemedicine technology for their practice after the pandemic.

**Table 1.** Physician demographics and practice characteristics.

Age, y	No. of physicians, %
≥35 to <45	58 (17.7)
≥45 to <55	79 (24.1)
≥55 to ≤65	123 (37.5)
>65	63 (19.2)
Annual no. of total hip replacements	
≥0 to ≤49	197 (60.1)
≥50 to ≤99	43 (13.1)
≥100 to ≤199	50 (15.2)
≥200 to ≤300	11 (3.4)
Annual no. of total knee replacements	
≥0 to ≤49	144 (43.9)
≥50 to ≤99	52 (15.9)
≥100 to ≤199	70 (21.3)
≥200 to ≤300	38 (11.6)

**Table 2.** Physician telemedicine visits before, during, and after the COVID-19 pandemic.

Percentage of total visits conducted by telemedicine	No. of physicians, %
Before pandemic	
0%	276 (84.1)
>0% to 5%	36 (10.9)
6% to 10%	4 (1.2)
11% to 20%	2 (0.6)
>20%	3 (0.9)
During pandemic	
0%	42 (12.8)
>0% to 5%	103 (31.4)
6% to 10%	63 (19.2)
11% to 20%	42 (12.8)
>20%	72 (22.0)
After pandemic	
0%	83 (25.3)
>0% to 5%	141 (43.0)
6% to 10%	47 (14.3)
11% to 20%	26 (7.9)
>20%	26 (7.9)

With each subsequent postoperative visit, surgeons were more amenable to conducting the visit via telemedicine: first postoperative visit (n = 128, 39.0%), second postoperative visit (n = 184, 56.1%), and third postoperative visit (n = 195, 59.5%). One-third of respondents would virtualize 2 visits within the first 90 days. Most of the physicians would be willing to do annual visits by telemedicine (n = 194, 59.1%). Ability to examine the patient (2.0 ± 1.0) was rated worse, overall, than either the experience using the technology (3.2 ± 1.0) or the capacity to communicate with the patient (3.6 ± 1.0). Daily step count, range of motion, and gait parameters, including cadence, stride, and walking speed, were frequently listed as important remote physiological monitoring tools for postoperative patients.

## Discussion

During the COVID-19 pandemic, we sought to better understand how orthopedic surgeons were using telemedicine technology. We were also interested in prior use and future use of the technology. Our findings demonstrate that orthopedic arthroplasty surgeons feel more comfortable conducting patient visits using telemedicine technology during routine follow-up than immediately after surgery. While most of the physicians did not use telemedicine as part of their practice prior to the pandemic, more than 1 in 5 physicians conducted over 20% of their practice using telemedicine during the pandemic. Despite the rapid adoption of telemedicine technology, nearly half of physicians noted they would not use telemedicine after the pandemic.

The use of telemedicine in orthopedics has been investigated by a number of other groups [3,10,17,20]. Recently, a randomized controlled trial by Buvik et al [3] investigating outcomes following video-assisted remote consultations and standard consultations found that there were no serious events related to the mode of consultation. In that study, at least 98% of both remote consultations and standard consultations were evaluated as either “good” or “very good.” To our knowledge, this is the first study that focused on the evaluation of telemedicine use from the perspective of arthroplasty surgeons.

A major postoperative concern is patient access and adherence to adequate and quality rehabilitation. A study published by Chughtai et al at the Cleveland Clinic, Cleveland, Ohio, held promising results in the realm of virtual physical therapy. The researchers evaluated various factors in telerehabilitation of patients who underwent total knee arthroplasty (TKA) or unicompartmental knee arthroplasty (UKA). Results showed that 78% of UKA and 76% of TKA patients adhered to the program. Overall, patients spent an average of 29.5 days doing postoperative therapy at an individual average of 26.5 minutes per day. The total time of postoperative therapy was 10.8 hours, with each patient performing an average of 13.5 different exercises [5].

A recent study analyzed whether a virtual-based intervention could improve clinical outcomes and thus reduce long-term health problems and associated costs, by resolving low physical activity. Patients were provided a generic accelerometer-based device and their progress was tracked over the course of ten 30-minute sessions spanning a 12-week period [14]. At our institution, we found that telehealth visits for physical therapy following total hip and knee replacements were effective and provided a promising alternative to in-person physical therapy following surgery [8]. In this limited subset of veterans, this study found that physical activity behavior change in combination with conventional rehabilitation protocols could be critical to resolving chronic poor physical activity and high costs following total knee replacement surgery [14]. In addition to reduced costs to patients and improved efficacy for clinicians, virtual postoperative rehabilitation may result in better adherence to physical therapy, allowing for more rapid and successful recovery following surgery [5,6,19]. This in part may be the result of enhanced monitoring of at-home rehabilitation, which allows physicians and therapists to modify or supplement therapy throughout the treatment regimen.

As health care continues to develop ways to decrease medical costs, telemedicine may be an alternative method to providing optimal patient care while reducing the total financial burden. Buvik et al [2] demonstrated that telemedicine consultations resulted in overall annual costs savings of 18,616 euros per 300 consultations. In the cost analysis, limitations included a lack of initiative on behalf of patients to apply for travel reimbursement, uncalculated production losses for individuals accompanying patients, and inability to account for the full costs of staff training. Furthermore, without time-consuming travel and the stress of navigating an institution, patients have reported feeling more at-ease in the comfort of their own homes, which may allow for more willingness and honesty in describing their condition and discussing their concerns. One major limitation of virtual medicine is the lack of a hands-on approach, which is essential in the proper evaluation of range of motion and soft-tissue, surgical site integrity. In addition, body language and facial expressions in response to pain or discomfort are important cues to providers but are often difficult to appreciate in virtual consults due to the inability to recreate pain-provoking movements. Another challenge may be a potentially low-quality audio-visual experience that hinders effective patient-clinician interaction. Finally, especially in the context of lower extremity arthroplasty, global assessment of the patient, including evaluation of gait and posture, is not as reliable on screen as in-person. On the contrary, given the bandwidth of communication across institutions and advanced imaging software, remotely obtained radiographs sent to the surgeon can be analyzed in real time during the virtual visit and serve as a crucial means of identifying fractures or assessing implant position regardless of where the patient is located.

While several drawbacks of telemedicine are simple inconveniences, some deficits—such as the increased likelihood of misdiagnosis—can have severe repercussions. For example, in their practice, Bluman et al [1] diagnosed a 65-year-old woman with arthritis of the right knee via a telemedicine visit. The patient subsequently fell at home, causing an oblique subtrochanteric fracture through a lytic lesion in the proximal femur. The contents of the lytic lesion were later analyzed by surgical pathology, which confirmed the diagnosis of metastatic thyroid carcinoma.

There are several limitations to this study. First, some data would have been more accurately recorded through direct, quantitative measurements. For example, rather than asking physicians to estimate the proportion of telemedicine visits they are conducting during the pandemic, our survey could have asked about the true proportion based on existing clinical data. However, attempts to collect accurate data on the true proportion of visits that were conducted as telemedicine visits likely would have resulted in substantial missing data due to nonresponse. Second, the physicians surveyed in this study may not have been representative of orthopedic arthroplasty surgeons, as a whole. Most of the respondents were from New York City, which was particularly hard-hit by the COVID-19 pandemic in its initial months [7,9]. Third, the types of orthopedic surgery practices within this population of physicians surveyed differed greatly. Some practices were completing 200 to 300 total hip and total knee replacements annually, whereas others were completing fewer than 50 of each procedure each year. The frequency of visits due to recent operative care likely influenced physician satisfaction with telemedicine.

In conclusion, our study of orthopedic arthroplasty surgeons demonstrates that while use of telemedicine technology was minimal prior to the pandemic, its use was widely adopted during the pandemic. Despite the shift in delivery of patient care, approximately half of physicians will continue to use telemedicine, and half will discontinue its use. It is thought that improvements in remote patient monitoring may make these nonadapting physicians more comfortable with telemedicine visits. While telemedicine will not take the place of in-person visits, it may play a role in chronic patient monitoring, such as long-term postarthroplasty visits in the future.

## Supplemental Material

sj-pdf-1-hss-10.1177_1556331620979984 – Supplemental material for Perspective of Attending Physicians on the Use of Telemedicine in an Outpatient Arthroplasty Setting During the COVID-19 Pandemic Click here for additional data file. Supplemental material, sj-pdf-1-hss-10.1177_1556331620979984 for Perspective of Attending Physicians on the Use of Telemedicine in an Outpatient Arthroplasty Setting During the COVID-19 Pandemic by Samuel A. Taylor, Joseph D. Lamplot, David A. Kolin, Kaitlin M. Carroll, Kevin Plancher and Fred Cushner in HSS Journal®: The Musculoskeletal Journal of Hospital for Special Surgery

sj-zip-2-hss-10.1177_1556331620979984 – Supplemental material for Perspective of Attending Physicians on the Use of Telemedicine in an Outpatient Arthroplasty Setting During the COVID-19 Pandemic Click here for additional data file. Supplemental material, sj-zip-2-hss-10.1177_1556331620979984 for Perspective of Attending Physicians on the Use of Telemedicine in an Outpatient Arthroplasty Setting During the COVID-19 Pandemic by Samuel A. Taylor, Joseph D. Lamplot, David A. Kolin, Kaitlin M. Carroll, Kevin Plancher and Fred Cushner in HSS Journal®: The Musculoskeletal Journal of Hospital for Special Surgery
