# Supplementary material for: Perspective of Attending Physicians on the Use of Telemedicine in an Outpatient Arthroplasty Setting During the COVID-19 Pandemic
Source: HSS J. 2021 Feb 21;17(1):31–5. doi: 10.1177/1556331620979984 (PMC8077992; doi:10.1177/1556331620979984)
Supplement: sj-pdf-1-hss-10.1177_1556331620979984 – Supplemental material for Perspective of Attending Physicians on the Use of Telemedicine in an Outpatient Arthroplasty Setting During the COVID-19 Pandemic [file sj-pdf-1-hss-10.1177_1556331620979984.pdf]

Telehealth Follow-up visits post-pandemic

1. 1. How many total hips/year?

   Mark only one oval.

   - 0-49
   - 50-99
   - 100-199
   - 200-300
   - > 300

2. 2. How many total knees/year?

   Mark only one oval.

   - 0-49
   - 50-99
   - 100-199
   - 200-300
   - > 300

3. 3. PRE-PANDEMIC, what percentage of your visits were conducted via telehealth?

   Mark only one oval.

   - None
   - <5%
   - 6-10%
   - 11-20%
   - > 20%

4. 4. DURING THE PANDEMIC, what percentage of your visits have been conducted via telehealth?

   Mark only one oval.

   - None
   - <5%
   - 6-10%
   - 11-20%
   - >20%

5. 5. Please rate your telehealth experience as it relates to technology?

   Mark only one oval.

	1	2	3	4	5	
Poor						Excellent

6. 6. Please rate your telehealth experience as it relates to your ability to examine the patient
?

Mark only one oval.

	1	2	3	4	5	
Poor	⬭	⬭	⬭	⬭	⬭	Excellent

7. 7. Please rate your telehealth experience as related to your ability to communicate with the patient?

Mark only one oval.

	1	2	3	4	5	
Poor	⬭	⬭	⬭	⬭	⬭	Excellent

8. 8. POST-PANDEMIC, what percentage of your follow-up visits do you expect will be conducted via telehealth?

Mark only one oval.

- ⬭ None
- ⬭ <5%
- ⬭ 6-10%
- ⬭ 11-20%
- ⬭ >20%

9. 9. When is your first post-op visit?

Mark only one oval.

- ⬭ 2-3 weeks
- ⬭ 3-4 weeks
- ⬭ 4-6 weeks

10. 10. Would you be willing to use telehealth for your first post-op visit?

Mark only one oval.

- ⬭ Yes
- ⬭ No

11. 11. When is your second post-op visit?

Mark only one oval.

- ⬭ 3-4 weeks
- ⬭ 4-6 weeks
- ⬭ 3 months
- ⬭ 6 months

12. 12. Would you be willing to use telehealth for your second post-op visit?

Mark only one oval.

- Yes
- No

13. 13. When is your third post-op visit?

Mark only one oval.

- 2-3 months
- 3-4 months
- 6-8 months
- 10-12 months

14. 14. Would you be willing to this third visit via telehealth?

Mark only one oval.

- Yes
- No

15. 15. Do you see the patient annually after 2 years?

Mark only one oval.

- Yes
- No

16. 16. Would you be willing to do annual visits via telehealth?

Mark only one oval.

- Yes
- No

17. 17. If objective, physiological remote monitoring data were available to you which parameters would you find most useful in managing post-operative patients? Select all that apply

Check all that apply.

- [ ] Daily step count
- [ ] Heart rate
- [ ] Range of motion
- [ ] Gait Parameters (cadence, stride length, walking speed, etc...)
- [ ] Blood Pressure
- [ ] Infection
- [ ] Micromotion
- [ ] Wound images

18. 18. What is your age?

Mark only one oval.

- 35-45 years
- 45-55 years
- 55-65 years
- >65 years

19. Please feel free to share any additional observations or comments

This content is neither created nor endorsed by Google.

Google Forms
